# Supplementary material for: Mitochondrial genomics of human pathogenic parasite Leishmania (Viannia) panamensis
Source: PeerJ. 2019 Jul 2;7:e7235. doi: 10.7717/peerj.7235 (PMC6611448; doi:10.7717/peerj.7235)
Supplement: File S1 [file peerj-07-7235-s001.docx]

**Mitochondrial genomics of human pathogenic parasite *Leishmania* Viannia *panamensis***

Daniel Alfonso Urrea, Omar Triana-Chavez, Juan Fernando Álzate

**Supplementary File 1, Table 1.** Sequence of primers designed and used to close internal gaps and the ends of the maxicircle kDNA of *L. panamensis*.

| **ID** | **Sequence** |
| --- | --- |
| CLOSE_MAXI_F | AACAAATCCCCAACAATCACAAATA |
| CLOSE_MAXI_R | GGCTACAACTAATCCCATCCA |
|  |  |
| MAXI_2_F | TTTTTATGTATGGATACACGTTTTG |
| MAXI_2_R | CCTTTTCTTTCCTCTCCGCTT |
|  |  |
| MAXI_5_F | TGACATTTTGTGGATTGCGTA |
| MAXI_5_R | CATAATCAAACACCAATCACTGGC |
|  |  |
| MAXI_3_F | TTTTTATGTATGGATACACGTTTTG |
| MAXI_3_R | CCTTTTCTTTCCTCTCCGCTT |
|  |  |
| MAXI_8_F | TGAGATTTTGATTTAGGTTTTGTGA |
| MAXI_8_R | CCAGTAAATTTATCAGGTACTGCTTTT |
|  |  |
| MAXI_11_F | TGTGCCAGTGATTGGTGTTT |
| MAXI_11_R | AACCAGTAAATTTATCAGGTACTGCTT |
|  |  |
| MAXI_13_F | TGATAAAATTTTACCTGAATGG |
| MAXI_13_R | CACCAACTAAAGGGGTACTA |
|  |  |
| CLOSE_GAP1_F | AGGAATTTTATTTGGTTTTT |
| CLOSE_GAP1_R | TTTTAATTTCTAGTAGATTTGGA |
|  |  |
| CLOSE_GAP2_F | TTTGATTGAcATTTTATTGTAATGG |
| CLOSE_GAP2_R | TTCTCAACCACAGTATGCTGTTAAA |

**Supplementary File 1, Table 2.** A. List of the 21 minicircles of an infective strain of *L. panamensis* assembled in the present work. B. Comparison of the minicircles of *L. panamensis* assembled in 1999 by Sanger sequencing and the minicircles assembled in 2017 by 454 reported in the present work.
